# A career ladder professional development approach to employee engagement for technicians in academic veterinary medicine

**DOI:** 10.3389/fvets.2025.1483926

**Published:** 2025-04-28

**Authors:** Kendra Fletcher, Valerie Osland Paton, Stephanie J. Jones, Jonathan M. Levine, Sharon C. Kerwin, Stacy Eckman

**Affiliations:** ^1^Department of Small Animal Clinical Sciences, Texas A&M University, College Station, TX, United States; ^2^Department of Educational Psychology, Leadership, and Counseling, Texas Tech University, Lubbock, TX, United States; ^3^School of Veterinary Medicine, University of Wisconsin-Madison, Madison, WI, United States

**Keywords:** career advancement, career ladder, employee engagement, veterinary medicine, veterinary technician

## Abstract

**Introduction:**

This qualitative case study explored how a career ladder advancement program, with programmatic changes based on employee input and programmatic evaluation, related to employee engagement among veterinary technicians in a veterinary medical teaching hospital. While research regarding the implementation and outcomes of career ladders exists in multiple healthcare, private industry, and higher education contexts, there is no research pertaining directly to Veterinary Technicians within a revenue-generating organization affiliated with an institution of higher education, limiting the ability to address veterinary technician retention.

**Methods:**

Framed using Kahn’s theory of employee engagement, the study involved semi-structured interviews of 17 veterinary technicians to ascertain contributors and barriers to employee engagement through a structured career ladder program for promotion.

**Results:**

The study found that value and communication, patient care and teaching, and professional growth and development all contributed to engagement. In addition, organizational structure and perspective (goals, strategies and approach), as well as lack of support, were the key barriers to employee engagement.

**Discussion:**

Key recommendations from the study are building robust advancement programs, adopting a meaningful approach to communication, consider staffing and workload needs, and directly address issues of climate and culture within the organization.

## Introduction

**Figure 1:**
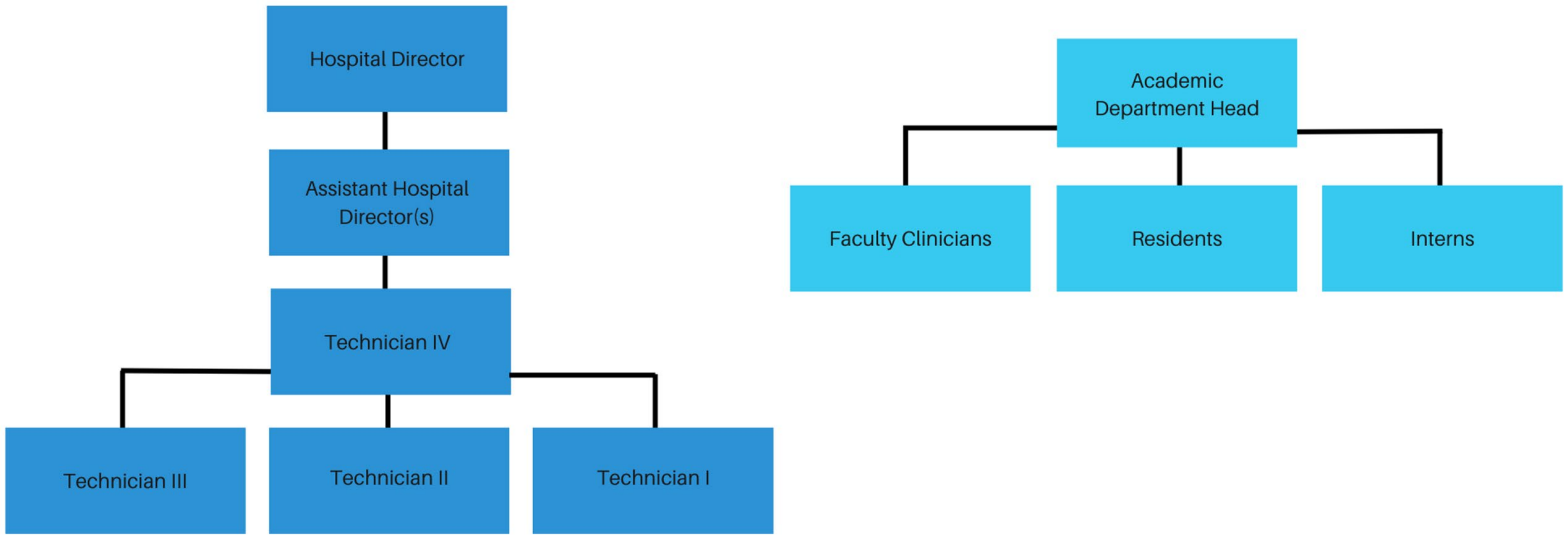
Simplified organizational chart noting reporting structure within the VMTH.

Multiple studies have found that employee engagement is correlated with employee retention and turnover intention widely across career fields (1–8), and specifically within the fields of healthcare (9–14) and higher education (15–18). Existing literature indicates a relationship between employee engagement and employee turnover among veterinary technicians (11, 19–21). For the purposes of this study, employee engagement is defined as “A psychological state involving harnessing employees’ ‘preferred self’ through physical, cognitive, and emotional energy during work role performances to promote connections with others, personal presence, and work tasks” (22) (Figure 1).

The study utilized Kahn’s (22) theory of employee engagement to determine the reported impact on employee engagement within the organization through the lens of advancement opportunities. This theory outlines dimensions of psychological conditions (meaningfulness, safety, and availability), which each incorporate experiential components, types of influence, and individual influences (Table 1). Kahn’s theory of employee engagement introduced the initial concept of employee engagement as measured by psychological conditions present or lacking for an employee which are impacted by intrapersonal, interpersonal, and system factors (1, 22–24) (Table 2). Kahn noted that employee engagement entails the cognitive, physical, and emotional energies that people express in their work roles. Kahn’s premise included that the measures of *meaningfulness*, *safety*, and *availability* could be used to determine the extent to which employees were engaged cognitively, physically, and emotionally.

**Table 1:** Kahn’s (22) grounded theory of employee engagement: dimensions of psychological conditions.

Dimensions	Meaningfulness	Safety	Availability
Definition	Sense of return on investments of self in role performances	Sense of being able to show and employ self without fear of negative consequences to self-image, status, or career	Sense of possessing the physical, emotional, and psychological resources necessary for investing self in role performances
Experiential components	Feel worthwhile, valued, valuable, feel able to give to and receive from work and others in course of work	Feel situations are trustworthy, secure, predictable, and clear in terms of behavioral consequences	Feel capable of driving physical, intellectual, and emotional energies into role performances
Types of influence	Work elements that create incentives and disincentives for investments of self	Elements of social systems that create situations that are predictable, consistent, and nonthreatening	Individual distractions that are preoccupying in role performance situations
Influences	Tasks: Jobs involving challenge, variety, creativity, autonomy, and clear delineation of procedures and goals	Interpersonal relationships: Ongoing relationships that offer support, trust, openness, flexibility, and lack of threat	Physical energies: Existing levels of physical resources available for investment into role performances
	Roles: Formal positions that offer attractive identities, through fit with a preferred self-image, and status and influence	Group and intergroup dynamics: Informal, often unconscious roles that leave room to safely express various parts of self; shaped by dynamics withing and between groups in organizations	Emotional Energies: Existing levels of emotional resources available for investment into role performances
		Management style and process: Leader behaviors that show support, resilience, consistency, trust, and competence	Insecurity: Levels of confidence in own abilities and status, self-consciousness, and ambivalence about fit with social systems that leave room for investments of self in role performances
			Outside life: Issues in people's outside lives that leave them available for investments of self during role performances

The figure demonstrates the details of Kahn’s (22) theory of employee engagement, highlighting the categories and subcategories of employee engagement. Adapted from Psychological conditions of personal engagement and disengagement at work, by Kahn’s (22). Copyright by Academy of Management Journal. Reprinted with permission. These dimensions relate to employee personal engagement or disengagement and are expressed through expression and employing factors (Table 2). Kahn (22) describes personal engagement and disengagement as the extent to which people are present during moments of role performances. Kahn’s (22) described employee engagement and disengagement as being characterized by both employing factors (tasks) and expressive factors (identity, thoughts, and feelings). The components of each factor are described in Table 2. Disengaged employees perform roles based on external scripts rather than internally reflecting on and enacting the role as an invested participant.

**Table 2:** Kahn’s (22) grounded theory of employee engagement: employing and expressive factors of engagement and disengagement.

	Engagement	Disengagement
Employing factors (tasks)	Effort (94)	Automatic or robotic behaviors (96)
	Flow (95)	Burnout (97)
	Mindfulness (98)	Apathy/detachment (99)
	Intrinsic motivation (100)	Effortlessness (94)
Expressive factors (identity, thoughts, feelings)	Self-expression	Defense of self
	creativity (101)	defensiveness (102)
	use of personal voice (103)	Bureaucracy (104)
	emotional expression (105)	Emotional inexpressiveness (96, 105)
	authenticity (106)	self-estrangement (107)
	Non-defensive communication (108)	
	playfulness (109)	
	ethical behavior (110)	

The figure demonstrates the details of employing factors and expressive factors for Kahn’s (22) definitions of employee engagement and disengagement. Theoretical foundation sources are cited for each factor.

Utilizing Kahn’s theory allows for separation of general experiences and perception of the work tasks and engagement or disengagement within roles (e.g., happy with work overall, but unhappy in a supervisory role), to better understand the impact of the career ladder advancement model.

As a component of employee engagement career advancement and training opportunities have been found to correlate with increased levels of reported employee engagement (25–41). One approach is a career ladder advancement model, which outlines the education, skills, and experience necessary for career advancement in an organization (42–47). This model has been utilized in numerous fields, including healthcare (10, 42, 48–50) and higher education (44, 48, 51, 52). Researchers have explored the decision to implement a career ladder model to address voiced employee dissatisfaction and organizational efforts to improve engagement and the overall organizational climate (44–47). Career ladders have been noted as an opportunity to prepare employees for future advancement within the organization, and as a method to integrate employees’ work with the education, research, and service missions of the institution (2, 44, 48).

Despite anticipated growth in available veterinary technician positions, concerns about licensure status, pay rates, and acknowledgement of veterinary technicians’ contributions have created insecurity about the future of the field (11, 53, 54). High rates of turnover and burnout, as well as veterinary technician shortages in high-demand areas, have contributed to additional stressors on veterinary practices, including veterinary medical teaching hospitals (30, 54–60). These concerns also highlight the need for prioritizing professional development and employee engagement for Veterinary Technicians (11, 30, 53–58).

Several studies highlight the complexity of capturing multiple measures of individual skill, performance, and successful incorporation of organizational competencies (28, 36, 44, 45, 61) to provide equitable and satisfactory career and promotional opportunities to high-performing employees. These studies noted this complexity as a catalyst for the adoption of career ladders, which provided a structure to capture several measures across multiple domains of training, experience, and professional development.

The present study reviewed a career ladder model of advancement enacted within a Veterinary Medical Teaching Hospital (VMTH). The Veterinary Technician Career Ladder model for advancement was established in the late 1990s through extensive communication with the study institution’s Human Resources (HR) representatives and hospital leaders. *Veterinary Technician* (VT) is the overarching term for staff who provide clinical support services, directly managing patient care in collaboration with Veterinarians. Prior to the establishment of the career ladder, there was no formal process for promoting VTs. By creating a formal model, the VMTH hoped to recruit and retain high-performing VTs within their trained service area, promoting career growth for staff and providing a higher standard of care within the organization.

Previous studies and reports note that in addition to fair wages for education and experience, employers should offer opportunities for veterinary assistants and technicians to effectively leverage their skills, provide opportunities for teaching of other technicians as well as client education, and to develop learning paths and career plans for professional growth and development (11, 30, 53, 54, 57–59).

When considering the needs of veterinary technicians, it is important to note the intangible costs associated with this work. Veterinary technicians can experience high levels of burnout and compassion fatigue from working with abused, neglected, and terminally ill or injured cases (11, 30, 57–60). Further, interpersonal relationships, workload, work schedules, a lack of appreciation, unreasonable expectations, poor communication, toxic attitudes, and conflict all contributed to burnout and technician disengagement and contributed to patient care errors.

## Methods

A within-site instrumental case study was used to gain understanding of employee engagement in the context of the career ladder advancement program, primarily to build understanding of the effectiveness of implementation (44, 51, 62). The setting for this study was a large public institution within the Association of American Universities (AAU) with a veterinary medical teaching hospital (subsequently referred to as VMTH). Prior to data collection, the researcher received an exemption from the Human Subjects Review Board and the Institutional Review Board of the study institution. Participants included veterinary technicians (VTs) who have participated in the career ladder model of advancement within the study setting. Exclusion criteria included those who had not participated in the career ladder process or who were not employed in the VMTH at the time of interview. Technical staff participation was solicited through purposive sampling owing to the need to engage participants who have participated in the career ladder advancement program, and an attempt by the researcher to obtain data from technical staff across multiple hospital services and career levels. Participants were selected based on their willingness to be interviewed and their role in informing the research program and the nature of the study’s specific focus on the referenced institution. Interested participants were instructed to contact the researcher at the provided email address to arrange an interview. Upon receiving an email response, the researcher provided a structured email thanking the participant for their interest and included a SignUpGenius link with anonymized responses to select an interview time. Participants were provided with a copy of the interview protocol and study description (Appendix). The researcher received notice of submitted interview times through an email from SignUpGenius, and responded to the participants to confirm the date, time, and location of the face-to-face interview. At the time of the study, VMTH leadership estimated that 60 current veterinary technicians had achieved promotion through the career ladder program, and interviews were conducted with 17 participants meeting the inclusion criteria who responded to the interview invitation. Interviews were conducted in April 2022 (15 in person and 2 by Zoom at the request of the participants) and each lasted between 22 min and 70 min, using interview questions based on Kahn’s (11) theory of employee engagement. Interview questions built on Kahn’s semi-structured interview approach (11), with questions designed to address changes to experiences within the hospital over time, key contributors to technical staff engagement and disengagement, factors indicative of employee engagement or disengagement, and in what ways technician supervisors and the organization contribute to technical staff engagement and disengagement (Appendix – interview questions). Each of these questions served to address the study’s purpose to determine key factors related to technical staff engagement or disengagement pertaining to the existing technical staff career ladder. Pseudonyms have been used, with participant approval, to ensure anonymity in responses.

Data analysis began with automatic interview transcription through Otter.ai software, followed by manual review and confirmation by the researcher using the transcriptions and recordings from the interview. Completed transcriptions were then provided individually by email to each participant for member checking prior to further analysis. Thorough readings of interview notes and transcriptions provide a basis of understanding for the interview responses and considered the data quality to ensure accurate representation of the data to the greatest extent possible (62).

Deductive coding and inductive coding were used to determine key themes from the interview responses. Deductive coding is a method within thematic analysis that allows for identifying and analyzing themes in the context of an existing theory. Where data is. Inductive coding involves analyzing raw data to develop codes and themes, which contribute to establishing connections between concepts to identify relationships between themes and subthemes (24). Deductive coding was conducted using ATLAS.ti software, and then inductive coding took place utilizing this software and review of all transcript data by the researcher to identify key themes emerging from all responses. Initial coding assignments were made for the categories associated with Kahn’s (22) measures of availability (physical energy, emotional energy, insecurity, and outside life), meaningfulness (work interactions, task characteristics, role characteristics), and safety (interpersonal relationships, group and intergroup dynamics, management style and process, organizational norms).

Following deductive coding, inductive coding was completed by creating spreadsheets by each assigned code from the open coding phase, and manually reading each code and grouping similar codes into broader categories, regrouping data as needed to ensure accuracy and consistency. After broader categories were determined, the data was further synthesized into themes based indicated by key phrases, addressed in the results section below.

## Results

The emergent themes for this study have been organized into the broad headings of: (1) employee engagement contributors, (2) employee engagement barriers, and (3) employee engagement decisions in relation to the career ladder model of advancement to provide clarity in establishing an outline for the data. Subthemes are discussed beneath each heading.

### Employee engagement contributors

Analyses of the data produced three themes specific to employee engagement contributors: (1) value and communication; (2) patient care and teaching; and (3) professional development and growth.

#### Value and communication

As a subtheme, value and communication can be represented by employee voice, the willingness and opportunity to speak up and share thoughts, ideas, questions, and concerns (63, 64). Participant responses indicated that feeling valued contributes to their engagement, while feeling devalued hinders engagement. Further, the participants referenced ways in which having a voice relates to the career ladder advancement model.

Having a voice was noted by several VTs as a key contributor to their engagement. Cassie said,

With my clinicians, I feel confident in my ability and my position, that I can speak up and that if there’s something that I feel uncomfortable with or am wanting to know, that they’re not going to be like ‘why are you questioning me?’ They will understand”.

This sentiment was echoed by Molly, who said, “I’m able to be myself, and my service functions well with the dynamic we have between the Veterinary Technicians on the clinic floor”. The perception of value and communication in Veterinary Technician-Supervisor and Veterinary Technician-Administration interactions was mentioned by several participants. While this is related to leadership support, which will be discussed in the next section, it is also relevant to VTs’ feelings of value within the organization. Several VTs noted the importance of positive interactions with their supervisors. When asked about interactions with their supervisor, several VTs responded positively, saying “my boss is amazing” (Casey), “my supervisor is wonderful” (Cassie), and “my direct supervisor is very encouraging” (Willa). In her interview, Molly stated, “Even when I don’t feel valued by the institution, I feel valued by my supervisor”.

In his interview, James referenced engagement stemming from the encouragement of his supervisor to share ideas and propose solutions to problems. He indicated his approach of “thinking outside the box, knowing the rules and using that to my ability to then come up with a new approach to something,” addressing specific situations where he’s been able to improve operations through this use of voice with his supervisor.

#### Patient care and teaching

Several participants indicated that contributing technical skills in clinical tasks promotes engagement and creates a sense of personal fulfillment (Annie, Emily, Kayla, Matt, Morgan). These contributions included patient care (Annie, Cassie, Kayla, Morgan) and teaching (Emily, Jess, Kayla, Matt, Paige, Sam, Willa).

Participants referenced patient care and the commitment to their chosen career as a primary motivation to engage. Paige noted, “this is a career for me it’s not a temporary job.” Cassie mentioned “I can show my compassion and the love that I have for this profession” and “I love my job and the patients.” Annie said “I do love my job. And I love what I do…I’m still majorly invested in it.” Maria stated, “I love working with the faculty, the clients, and the patients.”

Several participants referenced technical skills as an antecedent to patient care, noting that their technical skillsets positively impact the care and safety that they provide to their patients. Casey, Cassie, Emily, James, Kelly, Matt, Morgan, and Paige all noted that their technical skills, education, and experience contributed to their desire to provide excellent patient care and safety. It was clear from talking with the VTs interviewed that passion for supporting veterinary care and treatment underlies engagement in their work, including in the areas of teaching and development.

Teaching professional (DVM) students, interns, residents, and fellow VTs in the clinical environment was referenced as a strong contributor to engagement. Nine participants referenced teaching as a primary contributor to engagement in their work (Annie, Casey, Jess, Kayla, Maria, Matt, Morgan, Paige, Willa). Annie described this passion, saying

I love teaching the students, I love having the students come through…and this is what it’s about for me.

In discussing her perspective on teaching, Maria pointed out the benefits of an academic setting:

I didn’t realize I had a love of teaching until I came here. I love working with the students, love sharing knowledge, love learning knowledge. If you’re open enough, it goes both ways…some days, instead of teaching, I learn from our students.

Maria’s statement highlights the bidirectional nature of teaching and learning that promotes engagement through both knowledge-sharing and building rapport. James and Morgan’s view of teaching aligned with this, focusing on relationship-building to guide others in managing stress in the clinical environment and building their confidence (James, Morgan).

Participants also noted enjoyment in teaching interns and residents, who have more clinical experience and are seeking to build specialized skills. In addition to clinical skills, participants referenced the enjoyment of teaching “soft skills,” noting the ability to teach interns and residents to adapt in real-time, combining their knowledge of veterinary medicine with the unique challenges posed in the full-time clinical environment (Cassie, Emily, Paige). In addition to understanding organizational policies and protocols, VTs often have historical knowledge of established patients or similar cases, Teaching these skills contributes to a well-rounded knowledge base for veterinary practice.

James, Maria, and Willa talked about the importance of navigating the structure and culture of the organization, understanding the clinical services and groups to be as efficient and effective as possible. Emily, Paige, and Sam referenced coaching and mentoring, teaching VTs by promoting professional growth. Emily stated her interest as “coaching and mentoring…I personally get great joy out of seeing them succeed.” Paige described her motivations, saying,

I love teaching. And I love empowering people, specifically Veterinary Technicians, to grow and helping them find the resources versus sitting, you know, stagnant and waiting for somebody to hand it to you.

Overall, patient care and teaching were indicated as primary motivations for engagement for all interview participants. This theme is strongly indicated by participant responses as a key contributor to their job satisfaction and daily engagement with their work roles. Participants indicated a sense of accomplishment in contributing to veterinary medicine by utilizing their passion for excellent patient care and educating various stakeholders with the same goals.

#### Professional development and growth

A common theme addressed by participants was a desire to pursue education, training, and advancement to hone or expand their skills throughout the course of their work (Emily, James, Kayla, Morgan, Paige).

One item addressed was a need to feel supported by leadership through sharing available opportunities, allowing time for training, and supporting requirements for degrees or certificates. Nearly half of the participants indicated a desire for organizational support of VTs’ interest in research, presentations, and publications. This included mentoring and guiding VTs in this area (Bailey, Kelly), highlighting VTs’ investment in knowledge-sharing (Annie, Emily, Paige), and considering authorship for VTs contributing to clinical trials (Bailey, Jess, Jordan, Kayla, Kelly, Willa). Annie referenced giving presentations at conferences as a representative of the VMTH, noting that it kept her engaged through her passion for teaching. Paige and Emily addressed how their research experience benefited patient care through building the skills to monitor patients and overall trends, seek existing research literature, determine best practices, and incorporate those into clinical operations.

Several VTs noted that participating in continuing education kept them engaged in learning new skills and was a positive aspect of the career ladder advancement model (James, Kayla, Maria, Molly, Willa). Molly summarized the concept in saying “that’s something that’s appealing about working in a teaching hospital…you are able to constantly have access to continuing education and skill development” (103–104). Continuing education was made more successful by the ability to participate in both online and in-person courses (James, Willa).

As a component of professional development, cross-training was seen by some participants as a contributor to engagement through skill-building, teamwork, and engagement, particularly if cross-training opportunities promoted interactions among services that frequently interact (Bailey, Cassie, Jess, Jordan, Kayla, Maria, Paige, Willa). Cross-training allows VTs to shadow within other service areas of the hospital to build additional technical skills. Participation in cross-training is dependent on supervisor approval, as is any continuing education opportunity taking place during work hours (Emily, James, Kayla, Morgan, Paige).

Bailey noted “I think that the cross-training is good, just to broaden your skills and experience…I feel like it definitely can help you perform your job a little better.” Kayla expanded on this by highlighting the opportunity that cross-training provides in taking learned skills back to a VTs’ home service to share with others. In her interview, Jordan noted,

Cross-training shows your ability to work with new team members, your camaraderie to help the hospital as a whole, and well as utilizing skills and bringing out those skills that you haven’t before.

Several VTs noted the ability to assist in other areas of the hospital as a primary motivation for cross-training (Cassie, Emily, Jess, Jordan, Maria).

Study participants perceived professional development and growth opportunities as strongly associated with engagement and noted organizational support for pursuing education and training as a factor in personal fulfillment and job satisfaction. This theme, along with the themes of (1) value and communication and (2) patient care and teaching comprised the components of key contributors to participants’ perceived engagement within their work role.

### Employee engagement barriers

Analyses of the data produced two themes specific to employee engagement contributors: (1) organizational structure and perceptions; and (2) lack of support.

#### Organizational structure and perceptions

**Figure 2:**
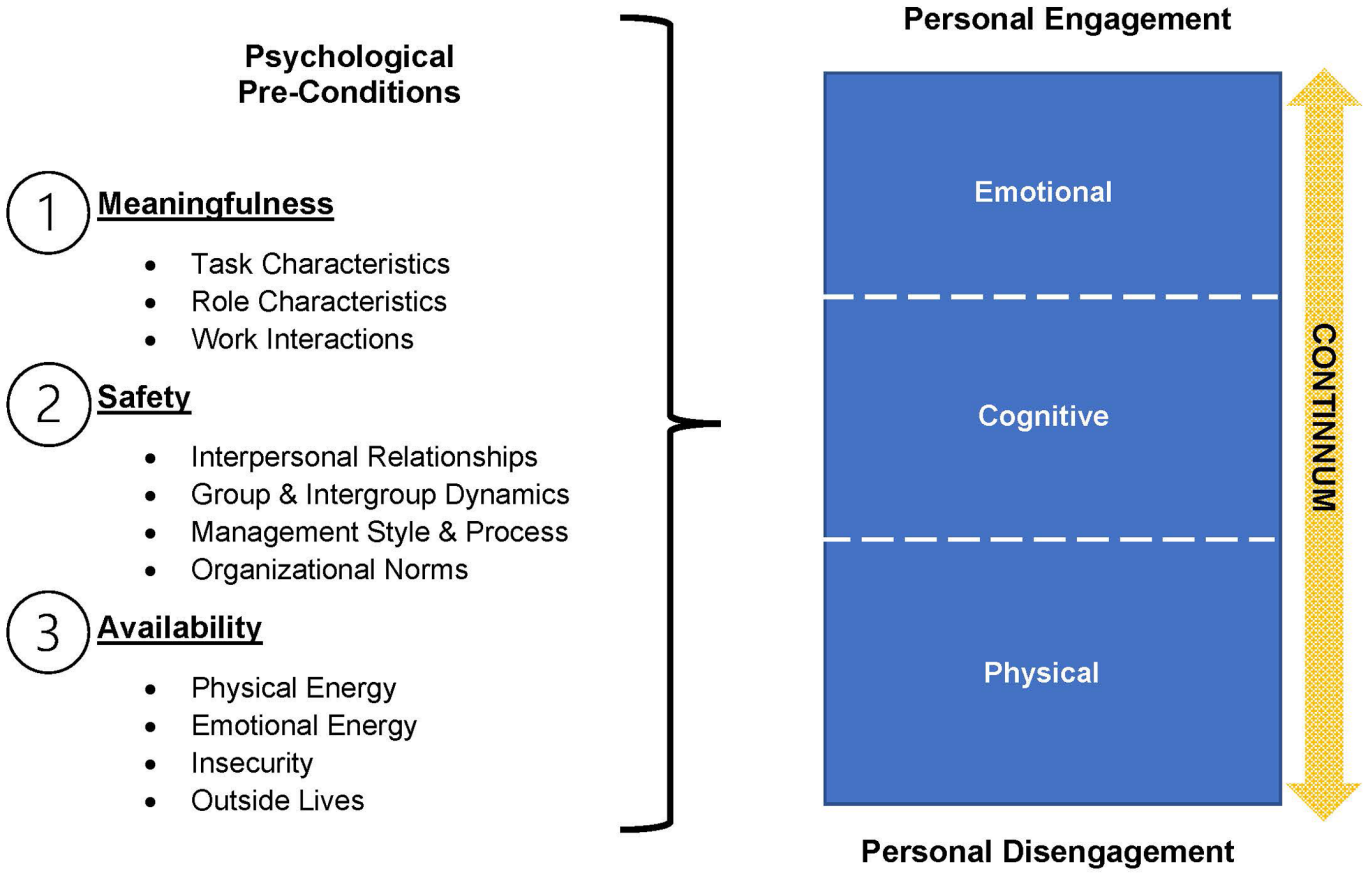
Illustrates Kahn’s psychological preconditions and their relationship to engagement or disengagement.

Many participants perceived a hierarchy within the VMTH that placed VTs below faculty clinicians, residents, interns, and veterinary students, limiting opportunities and inhibiting VTs from being heard as equal contributors to patient care (Jess, Jordan, Kelly, Morgan, Paige, Willa) (Figure 2). Some respondents indicated not feeling equally valued within the organization. This was summarized by Casey, who said,

I think that [Veterinary Technicians] don’t have much of a voice here. I think that the doctors tend to have a majority of the control. Which was shocking to go from a place where you have a lot of things under your thumb, and then you go to a place where they’re like, ‘Oh, you’re not allowed to do that.’ And really gaining trust from those doctors, residents, and interns, and being able to say, ‘I really can do that if you need me to.’

In contrast to those who indicated feeling supported and empowered by their supervisors, others noted a lack of support or positive rapport. These participants referenced supervisors’ lack of availability due to workload and job demands. Paige noted:

I don’t feel like my supervisor knows what I do on a day-to-day basis. And I don’t see her more than like a ‘hey, how’s it going’ kind of thing…I don’t necessarily know that it’s a fault on her part…it’s the way the system is.

As a supervisor, Emily noted a similar concern:

I feel bad because I can’t be on the floor all the time. And obviously you can’t be in two places at one time…if somebody needs just like a quick moment…you won’t be able to give them that time in the moment.

Maria noted the impact of supervisor unavailability, saying,

Whether it’s because of poor management or leadership skills, or because the supervisor’s just busy – whatever the reason, if I’m not helping educate you on what the opportunities are or how to get there…it’s a variable of both their experience and their perception.

Other VTs noted feeling a disconnect between themselves and administration, and a lack of understanding for the skills and experience of VTs working within the hospital (Cassie, Maria, Molly, Sam, Paige). Molly highlighted the dynamic between VTs and administration, saying,

I think there’s probably a rift between how it actually is on the clinic floor and how administration sees it…I think I center a lot of my feelings for things that are happening in administration that I feel like I can’t change, and then when I’m interacting with administration, I feel very rigid, and I wish that was not the case.

Cassie, who has worked at the hospital for over a decade, stated,

I’ve been here long enough that I don’t really have a problem speaking up or raising concerns…and I do feel like administration is making some positive changes to make improvement, but I feel like historically, there’s been a complete disconnect. They have no idea what we do. They don’t care what we do. They don’t care who we are.

One important note is that participants indicated optimism for change with the appointment of a new hospital director, which took place in 2021. Two VTs commented on this, saying “I am putting some trust, some faith in the hospital director…I think she values [Veterinary Technicians] more than our administration currently has” (Cassie) and “I am hopeful that the current administration seems to be making steps towards more accountability and more effort to improve [the] culture” (Maria). This perception could contribute to mitigating the barrier in this area.

#### Lack of support

As a research institution, conducting clinical trials is a component of patient care within the VMTH. In discussing VTs’ participation in clinical research, Bailey said:

We’re kind of encouraged to help and assist, but as far as being able to get on and be part of the publication itself, I don’t know how that would happen. Especially since some of these projects have already been going on for a bit longer than I’ve been here. So, everybody’s already been picked and assigned for what to do.

Jordan highlighted the competition resulting from this perception, describing how she was shown a task that needed to be completed to collect samples for a trial because she was the only VT available at the time. She indicated that this caused animosity from other VTs because “the brand-new person was able to ‘check something off.’ Literally, that was the wording.” She went on to say that she was co-author on a manuscript that had recently been submitted after a principal investigator (PI) approached her, knowing that research participation would benefit her preparation for veterinary school. “And it created some issues, regardless of the fact that it was for a very specific reason that others would not need.” Kelly provided additional perspective:

I like the ideas of publications for [Veterinary Technicians], but I don’t think that we’re really helped necessarily with that. I think it would be cool as a [Veterinary Technician] to be able to be more involved in research…I work at a teaching hospital, and I want to be part of these publications and help move the medical field forward.

In addition to continuing education, multiple VTs discussed their experience surrounding VT licensure and specialty certification. As discussed previously, avenues to veterinary technical careers include on-the-job training, licensure, and certification options. The VMTH, like many other institutions and private practices, employs on-the-job trained, licensed, and certified Veterinary Technicians (56, 65). In many states, a veterinary technician must have at least an associate’s degree and pass a licensing exam to become credentialed; titles for credentialed technicians vary by state. To obtain licensure in most states, VTs must have a field-relevant associate’s degree, complete required training, and pass a national licensing exam. These VTs’ credentials are noted as LVT (Licensed Veterinary Technician). In addition, some states allow technicians to become credentialed with on-the-job training and successful completion of the licensing exam. In referencing items related to LVTs, Annie and Paige both noted a lack of support in completing the necessary requirements for this process, specifically video recordings of them mastering hands-on clinical skills (Annie, Paige), with Paige noting,

I would come in and work 9- to 10-hour days and then I would come back in the evenings…with my dog to do my hands-on clinical skills and video them, because I had to do them here…I was not going to do venipuncture at my house.

Because an LVT program does not exist within the institution, these participants completed their training through an off-site program but felt unsupported in completing the requirements necessary to successfully obtain licensure.

In addition, some participants perceived a lack of support for their professional growth. When addressing the desire for additional training, Morgan explained that negative interactions with the supervisor responsible for approving training hindered her willingness to voice her requests:

We’re cordial, but I just try to avoid that where I can. And I think that’s kind of why I haven’t pushed so hard to find out about certifications, because that would be a barrier.

Others noted the perceived lack of support stemmed from time constraints, a lack of guidance, or apathy. Participants discussed experiences where supervisors were not responsive to requests for training or development opportunities, leaving VTs to seek out answers on their own (Kayla, Morgan) or attempt to coordinate their own training or education through other channels (Jess, Morgan, Paige).

Molly, despite a positive experience with her supervisor, noted concern over interactions she had seen among others:

I know [Veterinary Technicians] who are either are on the same level as me or lower levels, and their supervisors are not supportive or have actively sabotaged their attempts. And that’s really disheartening…if they have a supervisor that’s not engaging them or supporting them for whatever reason, does the career ladder board see that interaction, and is the supervisor protected because they’re the supervisor…And that’s not even because someone may or may not be qualified; it’s just if they have a personal vendetta against you, which unfortunately happens a lot on the clinic floor.

Participants also indicated that separation and a lack of support is reflected in conflict between service areas. Cassie highlighted this in saying,

I think it's great that we are specialized and have our specific skills. I would love to see this place be where we do work more as a team, because I feel like right now every service has their own agenda, and everybody works against each other. And that's not helping anybody. It's not helping the patient and it's not helping us. It's causing more tension between services, and it feeds down.

This perception was echoed by other VTs who felt that while specialization was necessary in the environment, it hindered engagement and collaboration with other VTs (Jordan, Kelly, Molly, Paige, Sam, Willa). Discussing technical skills and skill-building specifically, many participants indicated that specialization limited learning opportunities and negatively impacted the ability to gain knowledge and practice technical skills (Jess, Kelly, Molly, Morgan, Paige, Willa).

The impact of how these items manifested within the VMTH was summarized well by Maria, who stated:

And I think our biggest hindrance to people being able to progress and do things has more to do with our culture and with a lack of teamwork. That people want to set boundaries that don't have to be there.

### Employee advancement decisions related to the career ladder model of advancement

As the primary focus of the study, data collection and analysis served to build understanding of how employee perceptions of the current career ladder model contribute to VTs seeking career advancement within the organization. Analyses of the data produced two themes specific to employee engagement contributors: (1) categories and requirements; (2) organizational impact.

#### Categories and requirements

The main components in structuring the career ladder advancement model are the outlined categories that serve as guidelines for important areas for growth and organizational outcomes.

Regarding specific aspects of the career ladder, several VTs noted the difficulty experienced with the presentation category for the advancement packet. Willa noted, “They require public speaking…and that’s just not everyone’s forte. I mean, I’m terrified to talk in front of people. Terrified. And I do it because it was part of that requirement.” An additional aspect that contributed to VTs’ perception of voice and value was the letters of recommendation incorporated into the packet. Participants indicated that incorporating letters of recommendation contributed to feeling valued, with the only concern being that there is a limit of two letters of recommendation for each submitted advancement packet (Annie, Matt, Paige). Casey highlighted the importance of letters of recommendation for engagement, saying,

Having people that see the work you’re doing and recognize it and write that “I’ve seen her in the workplace. I’ve seen her attitude, her personality, her behaviors, everything, and she’s doing a great job.”

The honors and awards category was seen as hindering engagement because of the limited number of awards available and the perception that bias impacted awardee selection (Bailey, Jordan, Willa). At the time of the study, there were approximately 150 VTs in the VMTH and over 500 staff members total in the college, there are a total of 13 College-level awards, 6 University-level awards, 1 National Association of Veterinary Technicians in America (NAVTA), and approximately 10 VMTH-specific awards available each year (*Personal communication*). Many of these awards require a minimum of 2–5 years of service and can only be awarded to an individual once. While this does not account for additional industry, community, or private awards, participants viewed it as a limitation on technical staff who cannot obtain points for the awards category with such a small number available.

Several participants perceived that the requirements of the career ladder advancement packet hindered engagement by creating an environment of competition and scarcity that limited teamwork and collaboration. Specifically, the requirements related to the technical skills list and cross-training created the perception that teamwork took second priority behind meeting the requirements outlined in the career ladder. This was highlighted by consistent feedback that cross-training took VTs away from their service team, leaving the team short-handed (Bailey, Emily, Jess, Maria, Morgan, Paige, Willa). Casey summarized the issue in saying, “I have to leave my own service lacking because I have to make these hours happen at another area and be engaged over there.” This was exacerbated by the concern of service-specifics disproportionate impacts, where busier or smaller services experienced greater strain in allowing a VT time to cross-train in other services (Morgan, Willa). Several VTs indicated that the ability to address key skills was important, but the existing structure of the list created difficulties and disengagement (Casey, Cassie, Jordan, Kayla, Kelly, Matt, Molly, Morgan, Paige, Willa).

### Organizational impact

Several VTs discussed concerns over the requirements of the career ladder and the impact that these could have on daily clinical operations.

Within the current career ladder advancement model, supervisory responsibilities are required for promotion from a VT III to VT IV, and a points category for promotion from a VT II to VT III. Participants discussed barriers including not wanting to take on a supervisory role (Annie, Willa, Emily, Morgan), not having supervisory opportunities available (Cassie, Jess, Morgan) and in being supervised by others who might not be equipped for a supervisory role (Casey, Jordan).

For participants who were in either VT III or VT IV titles, needing to accept supervisory responsibilities was a consideration in the decision to seek advancement. Willa explained,

The way our career ladder is set, you go up and then you go into a supervisory role. Not necessarily that I wanted to be a supervisor, it’s just really the only way to advance. So, whether I wanted to be one or not, if I wanted to go up that’s all I could do.

Emily voiced,

I certainly think that there needs to be continued incentive and a continual career ladder for Veterinary Technician, but not maybe put that burden of ‘if you want to do [Veterinary Technician IV] then you now have to be a supervisor.

Within the organizational structure of the VMTH, a VT III has supervisory and administrative responsibilities within their existing team. These include coordinating with the VT IV to schedule service shifts, create guidelines and operating procedures, and provide training to other VTs. A VT IV is the primary supervisor of VTs within the service, and reports to an Assistant Director within the VMTH. Within the current structure, there is one VT IV assigned to a service or multiple services; “you cannot have two [Veterinary Technicians IVs] on the same management team” (Morgan). Further, promoting VTs requires that they are in a unit with a large enough team,

I have one service where it’s one doctor and one [Veterinary Technician] … she couldn’t be a Veterinary Technician IV in her just that one service. We would have to incorporate something else, the way ours are now because you have to be a supervisor. (Willa)

Cassie, Jess, and Morgan all noted a lack of available VT IV positions because of the supervisory structure,

I would go for [Veterinary Technician] IV, but [Veterinary Technician] IVs are supervisors in all the departments. So, if there’s not an empty Veterinary Technicians IV position to go to, you can’t go…I have something like 30 more years here and it feels like I’m stuck.

Jess similarly voiced,

The way they have it, it’s like once you hit Veterinary Technicians III and you’re still on the floor, there’s nowhere else for you to go. There’s no advancement…I’ve been stuck in this Veterinary Technicians III position for over 10 years.

Maria noted that completing the requirements for supervisory titles was difficult,

With supervisory training, sometimes it’s difficult to send [Veterinary Technicians] to some of the University pieces of training…you just kind of get creative and figure out what’s going to best suit them, so you have the least amount of difficulty.

This sentiment was echoed by Kayla, who noted that signing up for required in-person classes can be a struggle given the staffing needs within the clinic.

Another concern addressed for supervisory roles is whether completing training and the career ladder model suffice to determine supervisory skills and competencies. Casey voiced,

I know for the [Veterinary Technician III], it’s a supervisory level. And I just don’t think there’s any aspect in this that they can really tell if that person is a great leader or if that person’s going to do well with others and help them, and guide others and teach well.

She went on to say that this aspect of the career ladder fuels concern,

Because you see those people that shouldn’t be in a leadership position, but then they get the promotion, and the environment is more toxic. And those people who didn’t get it, or were thinking about going through the career ladder, now they don’t want to do it because they’ve seen who it promotes.

Jordan’s sentiment was similar,

There are leadership and supervisory aspects that come along with that, and not everybody is a born leader. Not everybody needs to be a supervisor or wants to be a supervisor. Or the opposite, they really want to be and probably shouldn’t…I’ve seen a lot of people get [Veterinary Technician III] that, I’m telling you, they’re a great [Veterinary Technician] but they cannot be a leader because they get way too emotionally involved.

Overall, the career ladder advancement model both impacts the organization and is impacted by it. Attitudes and behaviors related to the career ladder can affect the clinical environment, while advancement within the career ladder impacts the roles of staff within the VMTH. As a result, determining the interplay between these dynamics can be helpful in promoting engagement within the career ladder advancement model.

While participants noted numerous concerns with the career ladder advancement model, many also noted that they support the idea of requiring an advancement program in some capacity (Annie, Casey, Maria, Matt). “I definitely think there should be a plan in place for job growth and performance-building. Is it the career ladder? I do not know” (Morgan). Casey said,

I think it gives people something to work towards, something that keeps them engaged in the fact that there is the potential for that promotion. And you have to go through certain steps to get it. It’s not just handed to you.

The career ladder advancement model serves as a mechanism to promote growth and development among VTs within the VMTH and can provide clear goals and outlines in achieving advancement. Participant feedback indicates that aspects of the current model both contribute to and hinder engagement, but that a structured approach to advancement with consistent communication and requirements could have a positive impact on VTs and the organization.

## Discussion

The following discussion section addresses the themes and subthemes outlined in the results and their connection with existing literature.

### Employee engagement contributors

#### Value and communication

The theme of value and communication resonates with Kahn’s (22, 66) engagement measures of meaningfulness and safety. Meaningfulness is highlighted through work interactions, addressing employees’ need for relatedness (22) by building dignity, appreciation, respect, and positive feedback. Safety incorporates interpersonal relationships, specifically engaging in interactions from the authentic self (22, 66, 67). These aspects are built through the employing factors of personal voice, emotional expression, and nondefensive communication. For participants, these engagement measures manifested in interactions where their experience and thoughts were heard and valued. Working in a synergistic team environment promoted engagement through a sense of interpersonal belonging (16) and value (68, 69). These perspectives align with the findings of Sharafizad and Redmond (70), who noted the ability to share ideas and enact change as a contributing factor to employee engagement.

Participants’ relatedness needs were addressed through these interactions, contributing to meaningfulness, thereby promoting engagement. This echoes the findings of previous studies (8, 68–74) which found that communication and valuing employees positively impacted employees’ engagement in their work. Feeling valued and having a positive perception of their supervisor contributed to participants feeling safe in exhibiting their authentic selves at work (39, 41, 67, 71, 75).

#### Patient care and teaching

Patient care and teaching were contributing factors towards meaningfulness, specifically task characteristics and role characteristics. Task characteristics build a sense of meaning for employees by building competence through novel work and attainable challenges (67, 76, 77), while role characteristics represent identities that employees are given by the organization (22). Engagement is determined by how well this identity aligns with the individual’s self-identity (10, 13, 22, 66, 78, 79). Meaningfulness, defined as “receiving a return on investments of one’s self through physical, cognitive, or emotional energy” (22), is reflected in participants’ investment in patient care and teaching roles, resulting in a return on investment in the form of emotional and cognitive fulfillment associated with their passion for the career (17, 24, 67, 68, 80–82).

Participants attributed value to teaching and learning as opportunities to both invest in others’ knowledge and success and receive a return on investment through learning and building skills from the experience, which has been referenced in several studies on engagement (13, 17, 67, 69).

Specifically referencing patient care for veterinary technicians, Falley (30) and Sanders (59) both note the sense of identity derived from satisfaction and commitment to caring for patients. Sanders indicated that the sense of meaning derived from this work not only contributes to engagement but acts as a buffer mitigating job factors that would likely hinder engagement. Kane (11) referenced teaching as a contributor to high performance in his review of equine veterinary technicians, while Hayes et al. (57) and Kopp (83) noted a parallel for this among small animal veterinary technicians, referencing the daily level of hands-on care and interactions, and the ability to contribute to teaching high standards of patient care.

#### Professional development and growth

In this study, professional development and growth were characterized by management style and process (safety) and task characteristics (meaningfulness). Professional development can be defined as “increased learning and competence enhancement within a dynamic environment” (33). Safety can be established by experiencing leader behaviors that show support for employees, including openness and autonomy (40, 69, 84, 85) while task characteristics promote growth and learning (22, 24, 71, 86, 87). Participants’ desire for organizational support in pursuing training and education highlights the overlap between these two measures of engagement. Receiving leadership support when voicing interest in building knowledge and skills for professional growth promoted participants’ sense of safety in the organization, contributing to engagement (9, 68, 71, 88–90).

Given the context of the VMTH as a teaching hospital, participants felt that inclusion in clinical trials would promote engagement and professional growth, as well as patient care. This is supported by the findings of Leece and Jacquet (91), who concluded that keeping up with current research in a field promoted implementation of best practices and broadening knowledge of the field. Shelby^96^ also noted the positive impact of research participation for VTs on patient care and VT engagement.

The measure of safety was established by supervisors’ approach to professional development for staff. In studies surrounding staff engagement in healthcare and higher education fields, support for training and development from supervisors and organizational leadership was found to be a primary factor in engagement (17, 28, 29, 33, 40, 50, 68–71, 86, 87) federal government (7) private sector industry settings (9, 67, 76, 89).

### Employee engagement barriers

#### Organizational structure and perception

As barriers to engagement, the organizational structure and employees’ perceptions denote the absence of safety pertaining to organizational norms, and meaningfulness in relation to role characteristics. When an employee’s identity or behaviors contradict established organizational norms, anxiety and frustration can result (54, 76, 82). This directly relates to role characteristics, as individuals’ roles within an organizational system are dictated by organizational norms, and those roles determine accepted behavior (22, 23, 74).

Participants also perceived existing organizational norms separating VTs from administration and supervisors, with rigid boundaries that impede communication between the two groups. This limits openness and autonomy, minimizing VTs’ status and influence within the organization (24, 40, 69, 84, 85). Some participants noted that the existing organizational structure requires supervisors to oversee large numbers of VTs, making it difficult to allocate time to supporting their employees. These participants indicated experiencing a lack of support and guilt in making requests on their supervisor’s time. Participants also noted a perceived disconnect with administration, explaining that administrators’ separation from the clinic floor led to misconceptions and misunderstanding about daily operations that negatively impacted VTs’ trust in administration and belief that they were valued within the organization.

Several studies on staff engagement within IHEs noted that organizational structure and culture impacted employees’ perception of their value to the organization (15, 17, 68–70, 87). In particular, the environment created by senior administration and supervisors was found to impact meaningfulness and commitment among employees (17, 69, 87). Moore et al’s (60) study of veterinary technicians in private practice found that the physical absence or emotional disconnection of leaders hindered engagement by limiting communication and team cohesion, thereby decreasing meaningfulness related to the employees’ sense of value. This was echoed in other studies which found that organizational norms and employee engagement need to be owned by leadership and cultivated among employees for effective engagement (13, 67, 69, 72, 90).

#### Lack of support

Saks and Gruman (24) noted that “rewarding coworker and supportive supervisor relations were positively related to safety while adherence to coworker norms and self-consciousness were negatively related; and resources available were positive related to psychological availability while participation in outside activities was negatively related.” The engagement measures of management style and process (safety), group and intergroup dynamics (safety), and insecurity (availability) defined the lack of support experienced by participants in this study.

Management style and process manifest through a perceived lack of consistency, predictability, and openness from supervisors and administration towards VTs. This contributes to negative impacts for group and intergroup dynamics through variations in status and flexibility within and between units. As a result, some participants experienced insecurity in their status within the organization, subsequently exhibiting a lack of self-confidence and ambivalence that limited engagement, a concept that is supported within the employee engagement literature (8, 13, 66, 68–70, 72, 74, 76, 88, 90).

Studies on engagement have found that interpersonal dynamics within groups can attribute to stress or resilience, impacting employees’ sense of security within the work group (24, 57, 58, 60, 88). Further, limited resources and opportunities for professional development, negative citizenship behaviors in the organization, and high workloads and job stress are correlated with lower levels of employee engagement within IHEs (15, 43, 68, 70, 74, 86, 87).

Perceived lack of leadership support in pursuing growth and development opportunities negatively impacts employee engagement (8, 28, 29, 39–41, 81, 87). Encouraging the pursuit of further education and training within the bounds of the organization has been found to make employees more available to contribute to organizational outcomes (8, 15, 29, 33, 67, 70, 78) while providing time and resources for these endeavors promotes psychological safety and engagement among employees (4, 22, 24, 39, 40, 70, 82, 86, 90).

### Employee advancement decisions related to the career ladder model of advancement

#### Categories and requirements

Participant references to the impact of career ladder categories and requirements on employee engagement highlight all measures associated with availability (physical energy, emotional energy, insecurity, and outside life). Physical and emotional energy refer to individual capacity and energy levels that impact the ability to meet physical demands; emotional energy refers to available emotional resources compared to demand on those resources (22). Insecurity comprises an individual’s view of self, particularly in the areas of self-esteem, self-confidence, and ambivalence. Outside life is composed of the personal factors outside of the work environment that impact the resources available to engage at work. The tasks associated with advancement categories both added to and detracted from participants’ capacity in each of these areas.

Conference presentations and other external speaking opportunities are still limited despite growth in recent years (54), primarily in recorded online continued education lectures and national or specialty veterinary medicine conferences (56, 91). This condition negatively impacts employee engagement through the measure of availability by limiting the resources available to complete a task or outlined expectation (15, 24, 69, 78, 90). Full engagement in the work role requires individual capacity as well as channels through which that capacity can be channeled to achieve goals (17, 29, 39–41, 87).

#### Organizational impact

The relationship of organizational impact on employee engagement is best described through the measure of safety, specifically organizational norms and management style and process (22).

While participants referenced concerns regarding the career ladder advancement model, several supported the idea of requiring an advancement program for VTs in the VMTH. Participants believed that a structured plan for job advancement and professional growth was a necessary component to engagement and encouraged continued investment in individual development and contributions to the organization.

Studies have found that communication and decision-making were key factors in employees’ engagement within the organization (43, 69–71, 74, 75). Employees’ experience of bureaucratic barriers and lack of organizational fairness represented through a lack of communication contribute to disengagement (70, 74, 75). These issues represent a lack of open, honest, and transparent communication that promotes a sense of belonging and safety among employees, contributing to disengagement. In contrast, the experience of open and consistent communication contributes to meaningful engagement within the organization as it promotes a feeling that employees’ self-identity can be displayed using voice (10, 13, 46, 67, 78, 79).

### Limitations

This study was constrained by the small sample size and the limited scope of the study within one institution. While the estimated number of technicians meeting the inclusion criteria was 60, only 17 chose to be interviewed; the number of veterinary technicians who have been part of the career model and subsequently left the institution is unknown.

## Conclusion

Veterinary medical teaching hospitals fill an important role in providing veterinary medical care to client-owned animals while promoting the research and teaching missions of the affiliated institution of higher education. Within this context, VTs are critical to the success of these missions and the implementation of best practices in patient care. However, the veterinary technical field is facing concerns that include lack of recognition and career advancement, high stress and burnout, and structural inconsistencies. Studies recommended the development of structured approaches to professional development and advancement to address these concerns and contribute to the foundational growth of the veterinary technology career field.

The purpose of the study was to explore employee engagement in relation to a career ladder model of advancement among Veterinary Technicians employed within a veterinary medical teaching hospital affiliated with a large, public university located in the United States. Studying these employee engagement measures assisted in determining aspects of the career ladder model of advancement utilized within the study site are positively or negatively related to employee engagement to assist the organization in decision-making and implementation related to the career ladder model to address the needs of Veterinary Technicians.

The study findings support existing literature indicating a relationship between employee engagement and employee retention, including specific studies pertaining to higher education. It further supports the need for an established method to address employee advancement.

Through the perceptions of the participants, the overall findings of this study support that employee engagement among Veterinary Technicians within the study setting is impacted by factors associated with the career ladder advancement program. The participants’ experiences in career advancement at the study institution supported a greater understanding of the relationship between employee engagement and the career ladder advancement model in this context. For participants, a career ladder advancement model contributed to engagement through professional development, value, and encouraging participants’ commitment to veterinary medicine; in contrast, lack of support, organizational structure, and specific career ladder components hindered engagement for participants.

The study findings also support the relationship to Kahn’s engagement measures with employees’ perception of the advancement model and the overall organizational environment. Intentionality in building advancement models that address employees’ needs for safety, meaningfulness, and availability can contribute to increased engagement. Encouraging engagement by promoting employees’ investment of cognitive, physical, and emotional energy into their work helps to promote personal fulfillment for individual employees while contributing to organizational outcomes. Provision of a structured career advancement model can support opportunities for Veterinary Technicians to grow and advance, which promotes employee engagement while contributing to the organizational mission and the practice of veterinary medicine.

## Data Availability

The raw data supporting the conclusions of this article will be made available by the authors, without undue reservation.
