# Supplementary material for: A career ladder professional development approach to employee engagement for technicians in academic veterinary medicine
Source: Front Vet Sci. 2025 Apr 28;12:1483926. doi: 10.3389/fvets.2025.1483926 (PMC12067788; doi:10.3389/fvets.2025.1483926)
Supplement: Supplementary file 1 [file Table_1.DOCX]

# Appendix: Interview Protocol for In-Person Veterinary Technician Interviews

Interview Protocol Project: Determination of Veterinary Technician Engagement or Disengagement Related to a Career Ladder Advancement Program

Date of Interview:

Time of Interview:

Place of Interview:

Interviewer: Kendra Fletcher

Interviewee:

Pseudonym:

Thank you for agreeing to talk with me today. I appreciate your time and willingness to participate in this research study for use in my coursework. The purpose of this proposed study is to determine key factors related to technical staff engagement or disengagement pertaining to the existing technical staff career ladder advancement program. Of specific interest is feedback from Veterinary Technicians on engagement or disengagement in work, and the understanding of how the career ladder advancement program contributes to engagement or disengagement.

It is important that I let you know up front that your identity will be protected in this research with a pseudonym appointed in place of your name. Your pseudonym will be connected with your interview transcript, and at no time will your real name be shared with the data that you provide. Do you have a preference for the pseudonym that you would like to use? In addition, the institution’s name will not be noted in the results of the study but will be referenced as a veterinary medical teaching hospital affiliated with a large, public university in the United States.

As a reminder, your participation in this study is voluntary. Questions can be skipped, and we can stop the interview process at any time. If you do not have any questions about the study or the interview process, I will begin recording at this time.

Interview Questions:

1. In what ways do you feel you are able to bring your skills and experience into your work role?
2. In what ways do you feel that you are limited in bringing your skills and experience into your work role?
3. What aspects of your personality and identity do you feel that you are able to show to others while at work?
4. What aspects of your personality or identity, if any, do you feel you have to hide from others while at work?
5. What tasks or requirements for advancement, if any, do you feel take away from your engagement in your work?
6. What aspects of the current career ladder advancement program do you feel most strongly contribute to you being engaged in your work?
7. What aspects of the current career ladder advancement program do you feel hinder engagement with your work?
8. In what ways does the career ladder model of advancement help to keep Veterinary Technicians engaged at work?
9. How do you feel that interactions with your direct supervisor and other staff help or hinder your work towards advancement in the career ladder?
